# A Closed-Form Dual Quaternion Model for Drift Correction in TLS Pose-Circuits

**DOI:** 10.3390/s25237126

**Published:** 2025-11-21

**Authors:** Rubens Antonio Leite Benevides, Daniel Rodrigues Dos Santos, Luis Augusto Koenig Veiga

**Affiliations:** 1 Geomatics Department, Federal University of Paraná, Curitiba 81530-900, PR, Brazil; kngveiga@ufpr.br; 2 Military Institute of Engineering, Rio de Janeiro 22290-270, RJ, Brazil; daniel.rodrigues@ime.eb.br

**Keywords:** drift error, point clouds, laser scanning, dual quaternion interpolation, loop closure correction

## Abstract

**Highlights:**

Dual quaternion interpolation works better than the least squares method or decoupled interpolation of rotations and translations to distribute the closure error of 3D point cloud registration circuits.

**What are the main findings?**

- Linear dual quaternion interpolation is the most efficient solution for distributing the closure error along a circuit of 3D poses.
- Our CSI (Constant Smooth Interpolation) method unifies the translation and rotation correction of an entire circuit of poses in a single step.

**What are the implications of the main findings?**

- Easy implementation. It is possible to correct the closure error of the circuit just by linearly interpolating eight parameters.
- The interpolation can be performed fast and without losing the property of the shortest path on the manifold of the 3D poses.

**Abstract:**

Laser scanning allows for the rapid acquisition of three-dimensional data in the form of 3D point clouds. However, due to the accumulation of errors in the registration of multiple pairs of point clouds along the sensor’s trajectory, the generated 3D reconstructions exhibit drift, which creates global inconsistencies in the scan. To address this error, there are drift correction models that distribute the error along a closed circuit of stations. In this work, we present a model of this nature based on the linear interpolation of dual quaternions. This linear solution simultaneously refines rotations and translations in a closed trajectory without iterative computations or matrix decomposition. Experimental evaluations on eight TLS datasets indicate that the proposed drift correction model provides a robust average error reduction of 26%, with a maximum reduction of 41% in circuits with large drift. This simultaneous solution improves pose accuracy in closed trajectories with theoretical advantages that translate into efficient and fast implementation. Although validated using TLS data, the proposed pose-circuit correction model is sensor-agnostic and can be applied to other 3D mapping systems.

## 1. Introduction

Laser scanning transforms geoscience by enabling the rapid and precise acquisition of three-dimensional data. These precise 3D data are foundational for critical downstream tasks, such as environmental understanding via semantic segmentation [1] and object detection in autonomous driving [2]. The utility of all such applications, however, is contingent on the global geometric accuracy of the final, aggregated 3D model. Achieving this global consistency requires registering multiple point clouds, a process that invariably introduces cumulative drift error. While this is a general challenge in 3D mapping, it presents distinct difficulties when using Terrestrial Laser Scanners (TLSs).

This drift in TLS projects is compounded by three key factors. First, the point clouds have a non-constant, variable density due to the angular mode of acquisition. Second, exact 3D point correspondences cannot be found between scans. Most importantly, the operational strategy for TLS—maximizing the distance between stations to reduce fieldwork—is the primary driver of rapid drift growth. This wide spacing of several meters means that point clouds often overlap only in areas far from the scanner, where the point density is lowest. This sparsity, combined with the lack of exact 3D matches, degrades the quality of the pairwise registration, which depends on enough inlier matches. As a result, the sensor trajectory quickly deviates from its true path, accumulating significant errors with each successive station.

This work presents a linear Global Refinement Model (GRM) to correct this drift in a closed loop of TLS poses. The model uses dual quaternion algebra [3] to represent 3D rigid poses efficiently. This algebra unifies rotation and translation into a single mathematical object, the dual quaternion, which avoids the over parametrization found in matrices. Instead of using 12 parameters—3 for translation and 9 for rotation—it uses only 8 parameters, which is more efficient from a computational standpoint. In addition, dual quaternion algebra allows for faster and easier linear interpolation of 3D poses than with matrices. Specifically, our GRM applies the ScLERP technique [3] for drift correction in a closed loop of TLS poses, which is equivalent to the SLERP [4] for ordinary quaternions.

There are several GRMs for drift correction in TLS station graphs. Many of them include a consistency check of the graph edges. For example, in ref. [5], the authors refined a complete graph of TLS stations using two global optimizations. They first evaluated trajectory consistency with a loop closure check and then applied a minimum spanning tree to guide the second refinement with the algorithm of pose optimization described in [6].

In ref. [11], the authors refined pairwise registrations between a TLS-generated point cloud and 41 smaller clouds obtained through a Structured Light Scanner (SLS) using the model proposed in [12]. This model iteratively diffuses pairwise registration errors across the clouds. Similarly, ref. [13] employed an iterative graph diffusion method but parametrized transformations using dual quaternions. In [14], the authors proposed a method for the simultaneous registration of multiple TLS point clouds by globally adjusting rotations using quaternions and applying Singular Value Decomposition (SVD) on a hypermatrix, which follows the graph’s adjacency matrix.

In ref. [7], the authors used the Binary Shape Context (BSC) descriptor [8] to compute pairwise transformations for the TLS station graph. However, instead of performing an exhaustive search across all point clouds pairs as in [5,9], they aggregated the BSC descriptor during scan matching. This descriptor handles density variations effectively with a Gaussian kernel and achieves high computational efficiency by encoding information as binary vectors, enabling fast comparisons using the Hamming distance. The authors refined the final graph using the multi-view ICP algorithm proposed in [10].

In ref. [15], the authors introduced an iterative GRM based on graph structures, capable of handling the six degrees of freedom of pose transformations. This method generalizes the approach from [6]. Alternatively, closed-form solutions avoid iterative processes by using Spherical Linear Interpolation (SLERP) for loop-closure correction [4]. These methods backpropagate the closure error along the circuit, ensuring an identity pose in the closure of stations, as described in [16,17].

In addition to these classical approaches, hybrid systems are emerging that integrate deep learning with traditional graph optimization. For example, ref. [18] presented a LiDAR-SLAM system that uses conventional ICP for odometry and a factor graph for global optimization. They replaced the difficult task of place recognition with a module that learns feature descriptors for point cloud segments, enabling robust, viewpoint-invariant loop closures on a CPU.

These optimization principles have also been applied to correct drift in the field of deep learning-based visual odometry (VO). For instance, ref. [19] presented a hybrid, unsupervised VO network where pose drift was corrected by integrating classical pose graph optimization and bundle adjustment directly into the network’s training loop.

An example of an end-to-end approach is the work of [20], which used deep learning to replace the classical optimization process entirely, proposing PoGO-Net, a Graph Neural Network (GNN) designed to solve the complete pose graph optimization problem. Their method takes a noisy pose graph as input and learns to regress globally consistent poses. A key component of their network is a “de-noise layer” that learns to identify and drop outlier edges.

More recently, furthering the integration of deep learning and classical methods, ref. [21] presented ‘Wednesday’, a state-of-the-art multiway registration pipeline. This framework uses a learned, diffusion-based model for high-accuracy pairwise registration but employs a classical, non-learned algorithm for the core global rotation averaging. The authors note that recent learning-based graph optimization methods like PoGO-Net are difficult to reproduce or less effective. The pipeline then uses a novel, globally optimal translation re-estimation algorithm before applying a final diffusion-based network for joint pose refinement. This work highlights a trend of using deep learning to augment specific, non-robust steps while retaining the stability of classical geometric solvers for the core global optimization.

Many studies have addressed pairwise point cloud registration and global drift correction together. This research, however, focuses only on the latter. While pairwise registration is well-documented, correcting drift—a more complex problem that handles all 3D poses using GRMs—is less understood. Therefore, our primary contribution is a fully linear GRM that ensures convergence to the optimal solution without requiring iterations. This non-iterative quality is a desirable characteristic for such models [10].

Although existing works parameterize pairwise registration with dual quaternions [22] in a closed-form and iterative graph diffusion methods exist [13], to the best of our knowledge, no article has presented a linear, closed-form method for globally optimizing the poses within a point cloud loop.

## 2. Materials and Methods

### 2.1. Datasets

Table 1 lists our datasets collected using the Leica BLK360 TLS (Leica, Wetzlar, Germany), which were carefully selected with a realistic overlap to compare the proposed GRMs. Among the datasets, the UFPR dataset stands out as the most challenging to GRMs due to its total circuit length with 51 poses of the TLS. To obtain the initial approximated poses, we performed the pairwise registration in a coarse-to-fine manner using Fast Global Registration [23] and the Generalized ICP [24] implemented in a multiscale approach. Even with these two algorithms, about 15% of the pairs still could not be registered automatically in these cases, so we proceeded to manual registration and refined it with the G-ICP.

Table 1 lists the eight datasets used for validation. Four of these were not obtained by us: the Bremen (Dataset available at: https://kos.informatik.uni-osnabrueck.de/3Dscans/, accessed on: 9 November 2025) dataset, which was captured by Dorit Borrmann and Andreas Nüchter from Jacobs University, using Riegl VZ400 TLS (Riegl, Horn, Austria), and the Arch, Façade, and Courtyard datasets from [5], captured by a Faro Focus 3D TLS (Faro Technologies, Lake Mary, FL, USA). The Bremen and UFPR datasets are particularly notable, with circuit lengths exceeding 300 m. This extensive length is crucial as it amplifies the cumulative drift error, which helps to clearly visualize and quantify the behavior of the different GRMs.

**Figure 1:**
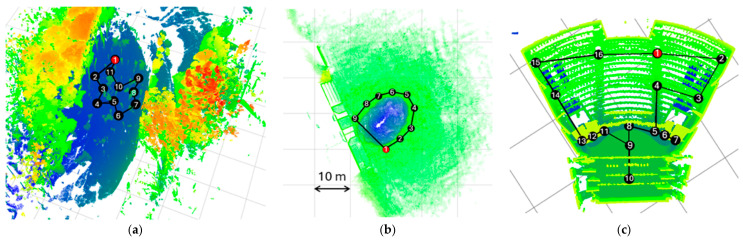

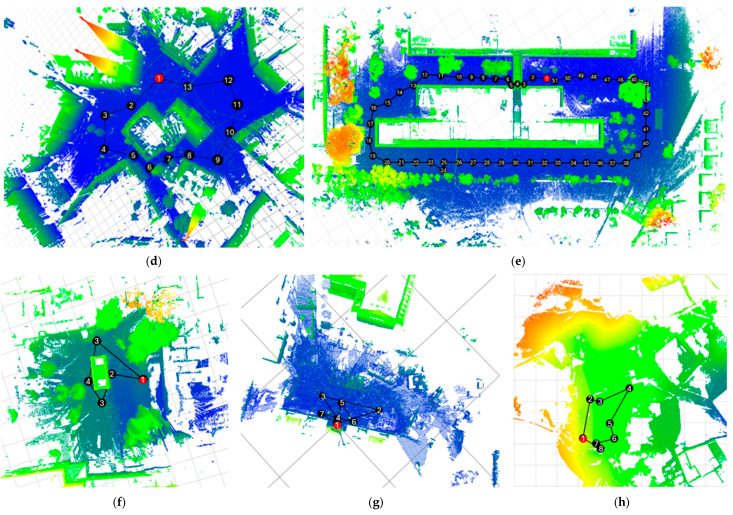
Datasets: (**a**) Wood; (**b**) Crater; (**c**) Theater; (**d**) Bremen; (**e**) UFPR; (**f**) Arch; (**g**) Façade; and (**h**) Courtyard.

Figure 1 provides a top view of the datasets: (a) Crater; (b) Wood; (c) Theater; (d) Bremen; (e) UFPR; (f) Arch; (g) Façade; and (h) Courtyard. Cover removed to visualize the stations in the Wood, Theater, and Façade datasets. The grid on the floor measures 10 × 10 m.

### 2.2. Proposed Global Refinement Method

Our Constant Smooth Interpolation (CSI) model refines translations and rotations simultaneously without any initial estimative, and does not perform iterations, matrix inversions, or decompositions, which means that it is linear and deduced in a closed form. We assume that the reader is familiar with the formulas to convert 3D poses to dual quaternions and vice versa, as well as the formulas for the exponentiation of dual quaternions. We recommend [25] for these formulations. The proposed loop closure correction operates in a closed circuit of n TLS stations $s_{1},\;s_{2},\ldots ,\;s_{n}$. Closed means the case where there is an overlap between the first and last cloud, so it is possible to estimate a transformation between them. Our GRM receives a set of relative poses and returns a set of optimized global poses, as follows:

$$GRM\left(relative\;poses\right)\to \;global\;poses,\;relative\;poses=\;\left[\;T_{0},T_{1\leftarrow 2},T_{2\leftarrow 3}\;\ldots ,\;T_{\left(n-1\right)\leftarrow n},\;T_{n\leftarrow 1}\;\right],\;global\;poses=\;\left[\;T_{0},T_{1\leftarrow 2},T_{1\leftarrow 3}\ldots ,T_{1\leftarrow n},T_{e}\;\right]$$

where a relative pose $T_{i\leftarrow \left(i+1\right)}=(R,t)$ is the rigid homogeneous transformation $T_{4\times 4}$ which transform the point cloud from station $s_{i+1}$ to $s_{i}$; thus, the relative poses originate from the pairwise registrations. The global poses are compositions of relative poses to the global origin, defined here as station $s_{1}$. Note that the first pose of both lists is the identity pose $T_{0}=I_{4\times 4}$, so in a closed loop with n stations there are n+1 relative poses and the same number of global poses.

The last item on the list of relative poses is the loop-closure pose $T_{n\leftarrow 1}$, which transforms the station $s_{1}$ to $s_{n}$. The last item of the global poses is the closure error $T_{e}$, which is the result of accumulating all relative poses. In an ideal scenario without drift, the composition of all relative transformations around a circuit should return to the starting point, therefore $T_{e}$ should equals $T_{0}$, but in practice the difference $\left|T_{e}-T_{0}\right|$ quantifies the closure error.

With these definitions we can start mapping all relative poses $T_{i\leftarrow \left(i+1\right)}$ to unit dual quaternions:

(1) $$r_{i}:=\;r_{i}\leftarrow T_{i\leftarrow \left(i+1\right)}$$

with i = 0, 1, 2,…, n+1, where the identity $T_{0}$ becomes $r_{0}$. Then, we compose the relative dual quaternions $r_{i}$ through the following sequence of multiplications:

(2) $$\begin{array}{c} {\vec{g}}_{0}=r_{0}\\ {\vec{g}}_{1}=r_{1}{\vec{g}}_{0}\\ \begin{array}{c} \vdots \\ {\vec{g}}_{n}=r_{n}{\vec{g}}_{n-1}\\ {\vec{g}}_{e}=r_{n}{\vec{g}}_{n} \end{array} \end{array}$$

In Equation (2) ${\vec{g}}_{i}$ is the global dual quaternion that maps the station $s_{i}$ to the global origin $s_{1}$, with $i=\left(1,2\ldots ,n\right)$. In Equation (2) ${\vec{g}}_{e}$ is the closure error in the dual quaternion form and $r_{n}$ is the loop-closure dual quaternion that maps points in station 1 to n. The right arrow is used to highlight the fact that we are composing poses in the direction defined earlier $i\to \left(i-1\right)$. Let us consider this the backward direction, since it is opposite to the sensor’s path. 

Nevertheless, in a closed circuit we can traverse the circuit in the reverse direction and express the transformations to reference each station back to $s_{1}$ using the inverse of the product in reverse order:

(3) $$\begin{array}{c} \begin{array}{c} {\overset{\leftarrow }{g}}_{1}={\left(r_{2}r_{3}\ldots r_{n-1}r_{n}\right)}^{-1}\\ {\overset{\leftarrow }{g}}_{2}={\left(r_{3},\ldots ,r_{n-1}r_{n}\right)}^{-1}\\ \vdots \\ {\overset{\leftarrow }{g}}_{n-1}={\left(r_{n-1}r_{n}\right)}^{-1}\\ {\overset{\leftarrow }{g}}_{n}={\left(r_{n}\right)}^{-1} \end{array}\\ {\overset{\leftarrow }{g}}_{e}={\left(r_{1}r_{2}r_{3}\ldots r_{n-1}r_{n}\right)}^{-1} \end{array}$$

as before, ${\overset{\leftarrow }{g}}_{i}$ is the global dual quaternion which maps station $s_{i}$ to the global origin $s_{1}$, with $i=\left(1,2,\ldots ,n\right)$, but in the reverse direction: $i\to \left(i+1\right)$. Let this be the forward direction. Note that in Equation (3) the last global dual quaternion is the inverse of the loop-closure dual quaternion: ${\overset{\leftarrow }{g}}_{n}=r_{n}^{-1}$; the number of accumulated poses in ${\vec{g}}_{i}$ is i, while in ${\overset{\leftarrow }{g}}_{i}$, it is n−i. This means that each new pose accumulated in one direction decreases a pose in the other direction, which is easily understood when walking in a closed circuit. Moreover, the closure error in the backward composition equals the inverse of the closure error in the reverse order: ${\overset{\leftarrow }{g}}_{n}={\left({\vec{g}}_{n}\right)}^{-1}$.

**Figure 2:**
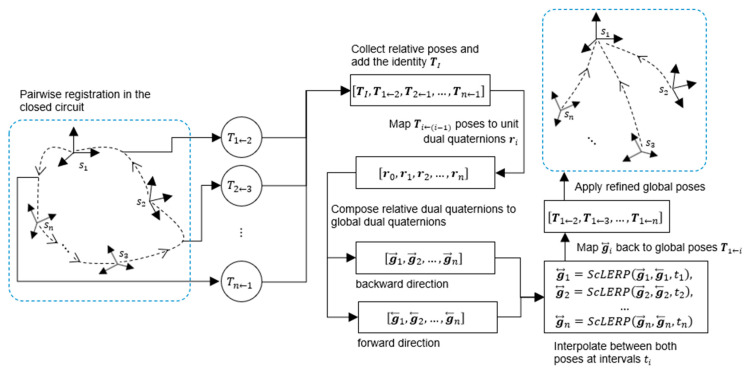
Pipeline of our proposed GRM for global pose optimization.

Let ${\overset{⃡}{g}}_{i}$ be the best estimative between ${\vec{g}}_{i}$ and ${\overset{\leftarrow }{g}}_{i}$. We define ${\overset{⃡}{g}}_{i}$ as follows: ${\overset{⃡}{g}}_{i}=CSI({\vec{g}}_{i},{\overset{\leftarrow }{g}}_{i},t_{i})$, where ${\overset{⃡}{g}}_{i}$ is the optimized dual quaternion that maps station $s_{i}$ to the global origin $s_{1}$, and $t_{i}$ is the interpolation interval $\in \left[0,1\right]$ with $i=\left(1,2,\ldots ,n\right)$. CSI stands for Constant Smooth Interpolation, which has a formulation identical to the Screw Interpolation (ScLERP) [3]. Figure 2 illustrates the pipeline of our GRM, culminating in the global refinement via ScLERP interpolation.

As shown in the figure, the pipeline begins with relative poses from pairwise registration, represented as 4 × 4 homogeneous matrices. These matrices are converted to dual quaternions, which are then accumulated to form global poses. This accumulation is performed in both the backward (Equation (2)) and forward (Equation (3)) directions of the circuit. The resulting forward and reverse global poses are interpolated using the optimal intervals $t_{i}$ and converted back to a final, refined set of homogeneous matrices. These final matrices represent the optimized global poses, which are applied to their respective point clouds to transform the entire circuit into the single coordinate system of the first station $s_{1}$.

From this point on, equations are developed to determine the optimal interpolation intervals. The ScLERP exponential formulation is presented in Equation (4), as follows:

(4) $$\begin{array}{c} {\overset{⃡}{g}}_{1}={\vec{g}}_{1}{\left[{\left({\vec{g}}_{1}\right)}^{-1}\;{\overset{\leftarrow }{g}}_{1}\right]}^{t_{1}}\;\\ {\overset{⃡}{g}}_{2}={\vec{g}}_{2}{\left[{\left({\vec{g}}_{2}\right)}^{-1}\;{\overset{\leftarrow }{g}}_{2}\right]}^{t_{2}}\\ \begin{array}{c} \vdots \\ {\overset{⃡}{g}}_{n}={\vec{g}}_{n}{\left[{\left({\vec{g}}_{n}\right)}^{-1}\;{\overset{\leftarrow }{g}}_{n}\right]}^{t_{n}} \end{array} \end{array}$$

to deduce the interpolation intervals $t_{i}$, one should write Equation (3) as follows:

(5) $$\begin{array}{c} {\overset{\leftarrow }{g}}_{1}={\vec{g}}_{1}{\left({\vec{g}}_{e}\right)}^{-1}\;\\ {\overset{\leftarrow }{g}}_{2}={\vec{g}}_{2}{\left({\vec{g}}_{e}\right)}^{-1}\\ \begin{array}{c} \vdots \\ {\overset{\leftarrow }{g}}_{n}={\vec{g}}_{n}{\left({\vec{g}}_{e}\right)}^{-1} \end{array} \end{array}$$

Equation (5) can be verified by replacing ${\vec{g}}_{e}$ with the product of the relative dual quaternions $r_{i}$. Substituting Equation (5) in Equation (4) we have:

(6) $$\begin{array}{c} {\overset{⃡}{g}}_{1}={\vec{g}}_{1}{\left[{\left({\vec{g}}_{1}\right)}^{-1}\;{\vec{g}}_{1}{\left({\vec{g}}_{e}\right)}^{-1}\right]}^{t_{1}}={\vec{g}}_{1}{\left({\vec{g}}_{e}\right)}^{-t_{1}}\\ {\overset{⃡}{g}}_{2}={\vec{g}}_{2}{\left[{\left({\vec{g}}_{2}\right)}^{-1}\;{\vec{g}}_{2}{\left({\vec{g}}_{e}\right)}^{-1}\right]}^{t_{2}}={\vec{g}}_{2}{\left({\vec{g}}_{e}\right)}^{-t_{2}}\\ \begin{array}{c} \vdots \\ {\overset{⃡}{g}}_{n}={\vec{g}}_{n}{\left[{\left({\vec{g}}_{n}\right)}^{-1}\;{\vec{g}}_{n}{\left({\vec{g}}_{e}\right)}^{-1}\right]}^{t_{n}}={\vec{g}}_{n}{\left({\vec{g}}_{e}\right)}^{-t_{n}} \end{array} \end{array}$$

now, one forces the triple composition constraint ${\left({\overset{⃡}{g}}_{i}\right)}^{-1}r_{i}{\overset{⃡}{g}}_{i-1}\approx r_{0}$, as follows:

(7) $$\begin{array}{c} {\left({\overset{⃡}{g}}_{1}\right)}^{-1}r_{1}{\overset{⃡}{g}}_{0}\approx r_{0}\\ {\left({\overset{⃡}{g}}_{2}\right)}^{-1}r_{2}{\overset{⃡}{g}}_{1}\approx r_{0}\\ \begin{array}{c} \vdots \\ {\left({\overset{⃡}{g}}_{n}\right)}^{-1}r_{n}{\overset{⃡}{g}}_{n-1}\approx r_{0}\\ {\left({\overset{⃡}{g}}_{e}\right)}^{-1}r_{n}{\overset{⃡}{g}}_{n}\approx r_{0} \end{array} \end{array}$$

The constraint forces ${\overset{⃡}{g}}_{e}$ equal the identity. ${\vec{g}}_{0}$ and ${\overset{⃡}{g}}_{0}$ also equal the identity, we have written these terms only for consistency in the equations, and they are left out in the next ones. Expanding the left side of Equation (7) using Equation (6) we obtain:

(8) $$\begin{array}{c} {\left[\;{\vec{g}}_{1}{\left({\vec{g}}_{e}\right)}^{-t_{1}}\right]}^{-1}r_{1}{\vec{g}}_{0}\approx r_{0}\\ {\left[\;{\vec{g}}_{2}{\left({\vec{g}}_{e}\right)}^{-t_{2}}\right]}^{-1}r_{2}\;{\vec{g}}_{1}{\left({\vec{g}}_{e}\right)}^{-t_{1}}\approx r_{0}\\ \begin{array}{c} \vdots \\ {\left[\;{\vec{g}}_{n}{\left({\vec{g}}_{e}\right)}^{-t_{n}}\right]}^{-1}r_{n}{\vec{g}}_{n-1}{\left({\vec{g}}_{e}\right)}^{-t_{n-1}}\approx r_{0}\\ r_{n}{\vec{g}}_{n}{\left({\vec{g}}_{e}\right)}^{-t_{n}}\approx r_{0} \end{array} \end{array}$$

now one can simplify Equation (8) by performing the inversion of the terms inside the brackets and replacing $r_{i}{\vec{g}}_{i-1}$ by ${\vec{g}}_{i}$ (see Equation (2)). The resulting terms cancel each other out as follows:

(9) $$\begin{array}{c} {\left({\vec{g}}_{e}\right)}^{t_{1}}{\left({\vec{g}}_{1}\right)}^{-1}{\vec{g}}_{1}={\left({\vec{g}}_{e}\right)}^{t_{1}}\approx r_{0}\\ {\left({\vec{g}}_{e}\right)}^{t_{2}}{\left({\vec{g}}_{2}\right)}^{-1}{\vec{g}}_{2}{\left({\vec{g}}_{e}\right)}^{-t_{1}}={\left({\vec{g}}_{e}\right)}^{t_{2}}{\left({\vec{g}}_{e}\right)}^{{-t}_{1}}={\left({\vec{g}}_{e}\right)}^{t_{2}{-t}_{1}}\approx r_{0}\\ \begin{array}{c} \vdots \\ {\left({\vec{g}}_{e}\right)}^{t_{n}}{\left({\vec{g}}_{n}\right)}^{-1}{\vec{g}}_{n}{\left({\vec{g}}_{e}\right)}^{-t_{n-1}}={\left({\vec{g}}_{e}\right)}^{t_{n}}{\left({\vec{g}}_{e}\right)}^{{-t}_{n-1}}={\left({\vec{g}}_{e}\right)}^{t_{n}{-t}_{n-1}}\approx r_{0}\\ {\vec{g}}_{e}{\left({\vec{g}}_{e}\right)}^{-t_{n}}={\left({\vec{g}}_{e}\right)}^{1-t_{n}}\approx r_{0} \end{array} \end{array}$$

with the product of powers (same base) we can add the exponents on the right-hand side of Equation (9) as follows:

(10) $$\begin{array}{c} t_{1}=0\\ t_{2}-t_{1}=0\\ \begin{array}{c} \vdots \\ t_{n}-t_{n-1}=0\\ 1-t_{n}=0 \end{array} \end{array}$$

The system in Equation (10) has n+1 equations and n unknowns, so it is possible to apply the Least Squares (LS) method to obtain an optimal solution. Let J be the Jacobian matrix of that system concerning the variables $t_{1},t_{2}\ldots ,t_{n}$; and b=[0,0,…0,1] the vector defined by the right-hand side of Equation (10). One can obtain the interpolation intervals $\tau =\left[t_{1},t_{2}\ldots ,t_{n-1}\right]$ by:

(11) $${\tau =\left(J^{T}J\right)}^{-1}J^{T}b$$

The result of Equation (11) is a function of the number of stations, in a closed circuit of *n* vertices, and τ is previously known, since the matrix J and the vector b follow a simple pattern:

(12) $$J={\left[\begin{array}{ccccc} 1 & 0 & \ldots & 0 & 0\\ -1 & 1 & \ldots & 0 & 0\\ \vdots & \vdots & \ddots & \vdots & \vdots \\ 0 & 0 & \ldots & -1 & 1\\ 0 & 0 & \ldots & 0 & 1 \end{array}\right]}_{\left(n+1,n\right)};\;b={\left[\begin{array}{c} 0\\ 0\\ \vdots \\ 0\\ 1 \end{array}\right]}_{n}$$

For a circuit with n = 4 stations, τ is:

(13) $$\tau =\left[\frac{1}{4},\frac{2}{4},\frac{3}{4}\right]$$

The LS solution divides the interval $\left[0,1\right]$ into complementary parts, so that $t_{i}+t_{n-i}=1$. This means that the CSI proceeds as a weighted mean between ${\vec{g}}_{i}$ and ${\overset{\leftarrow }{g}}_{i}$ where the weight is the index distance from station 1 given by t=i/n. In our example with n=4, one of the three interpolations happens exactly at ½, because going in the reverse or forward direction accumulates the same number of poses up to the middle of the circuit. As the same amount of error is expected in ${\vec{g}}_{2}$ and ${\overset{\leftarrow }{g}}_{2}$, we have ${\overset{⃡}{g}}_{2}=CSI\left({\vec{g}}_{2},{\overset{\leftarrow }{g}}_{2},2/4\right)$; For the first pose, the interval is closer to zero: ${\overset{⃡}{g}}_{1}=CSI\left({\vec{g}}_{1},{\overset{\leftarrow }{g}}_{1},1/4\right)$, and for the last pose, the interval is closer to one: ${\overset{⃡}{g}}_{3}=CSI\left({\vec{g}}_{3},{\overset{\leftarrow }{g}}_{3},3/4\right)$.

## 3. Results

Our GRM, referred to as Constant Smooth Interpolation (CSI), can optimize any closed circuit of TLS stations. We compared it against three other linear models on the same circuit for each dataset. The three models were (1) the LLS model, which adjusts and enforces the closure of translations using the Linear Least Squares (LS); (2) the SLERP model, which interpolates rotation like the approach in [16]; and (3): the SLERP + LS model, which combines the previous two to adjust rotations and translations in two steps [17]. All four models are linear, but only ours performs a simultaneous adjustment of rotations and translations.

We quantified the drift error of one pose using the metric defined in Equation (14), as follows:

(14) $$error_{i}={\left\|t_{i}^{g}-t_{i}^{e}\right\|}_{2}$$

and we also present the Total Error ($E_{Total}$) of the circuit:

(15) $$E_{Total}=\sum_{i=1}^{n}error_{i}$$

the Mean Absolute Error (MAE):

(16) $$MAE=\frac{1}{n}\sum_{i=1}^{n}error_{i}$$

and the Root Mean Squared Error (RMSE):

(17) $$RMSE=\sqrt{\frac{1}{n}\sum_{i=1}^{n}{\left(error_{i}\right)}^{2}}$$

where $error_{i}$ is the Euclidean distance in translation between the estimated global pose ($t^{e}$) and the ground truth pose ($t^{g}$). The ground truth of the global poses comes from the g2o model [26] incorporating all edges of the complete pose graph, which provides a higher quality reference than any of the GRMs and ensures reliable comparisons. We did not compare rotations because any change in these directly affects the translation vector of the pose.

**Figure 3:**
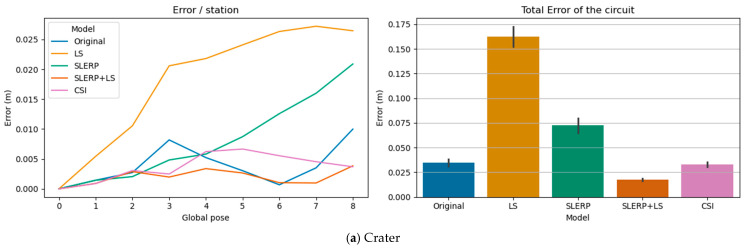

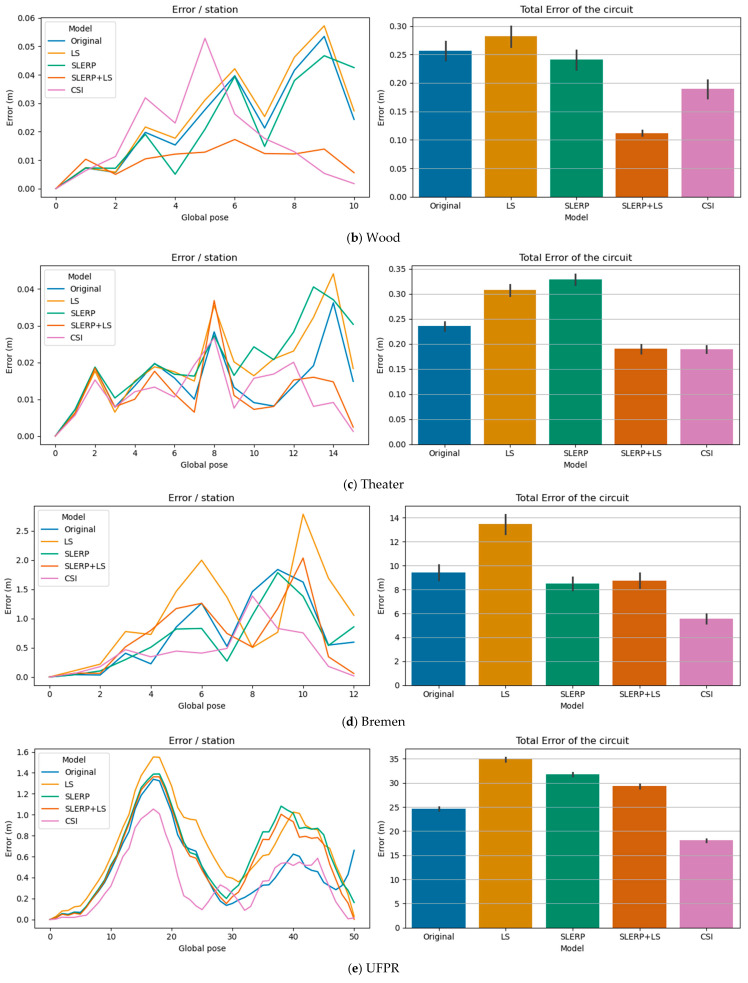

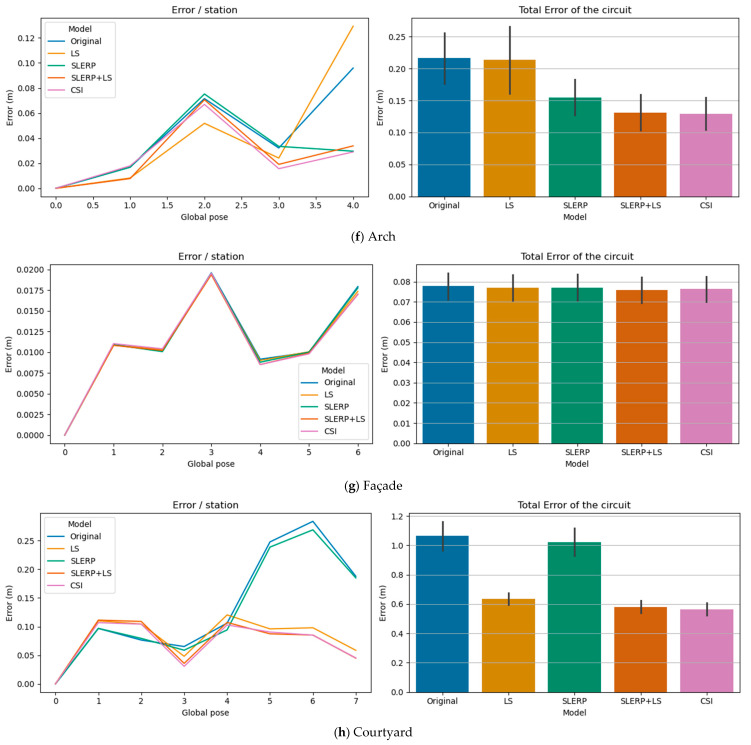
Error per station (left) and Total Error/model (right) of each circuit: (**a**) Crater; (**b**) Wood; (**c**) Theater; (**d**) Bremen; (**e**) UFPR; (**f**) Arch, (**g**) Façade, and (**h**) Courtyard. The total drift reduction in each dataset can be seen in the bars on the right, where the original error is reduced or increased by each GRM. The black bars in the figures on the right are the standard deviation of the circuit poses and represent how much the error varies throughout the circuit. The colors of the lines on the left correspond to the colors of the bars on the right where the smaller, the better.

Figure 3 compares the performance of each model across the eight datasets: (a) Crater, (b) Wood, (c) Theater, (d) Bremen, (e) UFPR, (f) Arch, (g) Façade, and (h) Courtyard. The line graphs on the left plot the error per pose, which originates at zero (the reference station) and accumulates along the circuit. The bar charts on the right summarize the Total Error for each model, with the error bars representing the standard deviation of the pose errors.

Table 2 quantifies the absolute error metrics for each GRM. It lists the Total Error (Equation (15)), which corresponds to the bar charts in Figure 3, along with the Mean Absolute Error (Equation (16)) and the Root Mean Squared Error (Equation (17)). Together, these metrics summarize the absolute error of each model for every circuit.

Table 3 quantifies the relative error for each GRM, expressed as a percentage. This value indicates the improvement (negative) or degradation (positive) of the Total Error relative to the original poses, calculated as: $relative\;error=-\left(1-{(E}_{GRM}/E_{Original})\right)$.

We can see in Figure 3 that adjusting rotations or translations in isolation is not a desirable alternative. The LS and SLERP models, when applied individually, showed inconsistent performance and, in several key datasets (Crater, Theater, and UFPR), delivered unsatisfactory results. Another disadvantage of the LS and SLERP models is the large variation in error across poses, which is shown by the standard deviation of the error, represented as a black bar in the bar graphs in Figure 3.

One can see in Table 3 that the LS model worsened the outcomes in five out of the eight datasets (Crater, Wood, Theater, Bremen, and UFPR). It showed negligible improvement in Arch and Façade and a 40% improvement in Courtyard. The limitations of the LS approach are well-known in the field of speleology, where loop closure corrections often degrade results rather than improve them, especially in the presence of blunders [27], which was not the case in any of the poses discussed here.

Similarly, the SLERP model degraded pose accuracy in the Crater, Theater, and UFPR datasets (Figure 3a,c,e) while it offered improvements in the other five datasets; these two GRMs (LS and SLERP) highlight the need for a model that works directly in the SE(3) space of 6D poses.

An alternative is to combine the output of the SLERP model (adjusted rotations) with the output of the LS model (adjusted translations). This is the proposal of the SLERP + LS model from [17], which delivered better results than our CSI model on the datasets with low drift: Crater (Figure 3a) and Wood (Figure 3b). Conversely, in datasets with large drift, Bremen (Figure 3d) and UFPR (Figure 3e), the CSI model delivered remarkably better results. In the remaining four datasets (Theater, Arch, Façade, and Courtyard), both models provided similar results, with CSI showing a minor advantage in three cases (Figure 3c,f–h).

The experimental results suggest that the primary advantage of the CSI model is its simultaneous handling of rotation and translation, which becomes critical as the magnitude of the error increases. In datasets with low cumulative drift (Crater and Wood), the rotational error was minimal. Consequently, the coupling between rotational and translational error is negligible.

The decoupled SLERP + LS model performed well here because its two-step process is a valid approximation. The SLERP step does not induce a significant new error, allowing the LS step to solve a clean translational problem. When the rotational error is close to zero, the coupling effect (the “lever arm” problem) is also close to zero. In this simplified scenario, the decoupled SLERP + LS model may even slightly outperform the CSI, as its LS component is, according to the Gauss–Markov Theorem [28], the Best Linear Unbiased Estimator (also called BLUE). Thus, assuming that the errors are uncorrelated, which is the case for the translational error since the movements in $R^{3}$ are independent, it is not surprising that the SLERP + LS method delivered good results when operating with a rotational error close to zero.

However, in long trajectories with large drift (Bremen and UFPR), the rotational and translational errors were significant and strongly coupled. A large rotational correction, when applied in isolation (as in SLERP), induced a substantial, new translational error across the circuit. As rotation and translation are not independent, a small rotational error at the beginning of a long 400 m circuit (like Bremen) will negatively affect the rest of the trajectory, creating a significant translational error at the end.

The SLERP + LS model failed because its first step contaminated the input for the second. The SLERP technique operates in the nonlinear $S^{3}$ manifold, while the LS technique operates in $R^{3}$, a linear space. This decoupled optimization process can be interpreted as an inadequate approximation of the shortest path between the 6-degree-of-freedom (6-DoF) poses, which describes a geodesic in the SE(3) manifold.

The CSI model excels in these high-drift scenarios precisely because its dual quaternion formulation treats the 6-DoF pose as a unified entity. It distributes the error along the true SE(3) geodesic, inherently modeling the nonlinear coupling between translation and rotation, thus providing a geometrically sound and more accurate correction.

**Figure 4:**
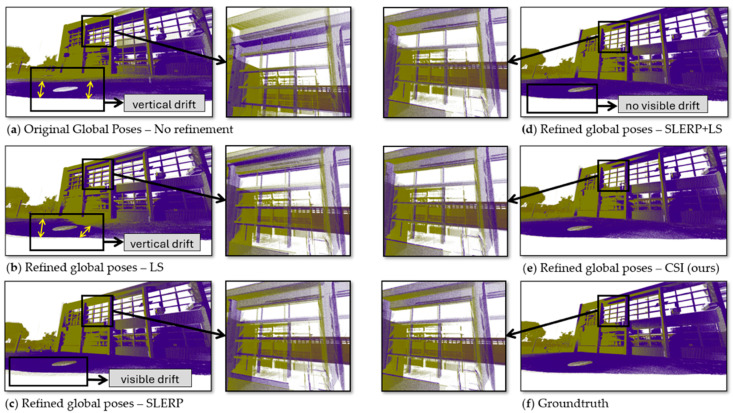
Effect of each GRM in the drift correction of the UFPR dataset. All scenes were taken from the same viewpoint. The blue cloud is from the beginning of the circuit, the green cloud is from the end of the circuit. (**a**) **Original Poses (No Refinement)**: Considerable vertical misalignment (drift) was visible between the initial (blue) and final (green) clouds. (**b**) **LS**: The model incorrectly shifted the green cloud downwards, and significant misalignment remained. (**c**) **SLERP**: Vertical alignment was improved, but horizontal misalignment became apparent between the window frames. (**d**) **SLERP + LS**: Drift was reduced, which was noticeable in the window frames, but vertical drift persisted in one of the clouds, although not visible. (**e**) **CSI** (Ours): The proposed model attenuated the vertical drift more effectively, yielding the closest reconstruction to the ground truth. (**f**) **Groundtruth**: In the groundtruth poses, there was no drift; the windows and pillars lined up perfectly.

In the Bremen dataset (Figure 3d), the CSI model delivered a 41% improvement over the original poses, significantly surpassing the 7% improvement in the SLERP + LS model. In the most extensive dataset, UFPR, the CSI reduced the error by 27% (Figure 3e), whereas SLERP + LS increased the error by 18% when compared to the original poses. Figure 4 depicts the qualitative result of each GRM in the closure of the UFPR circuit, where the effect of drift was considerable.

Figure 4a highlights significant drift along the Z-axis, evidenced by a pronounced vertical displacement between the initial (blue) and final (green) point clouds at the circuit’s extremities, particularly visible in roof structures. In Figure 4b, the drift was partially mitigated, but a detailed analysis revealed that the LS model threw the green clouds down—an expected outcome of the Least Squares (LS) model, as it does not account for rotational adjustments. Figure 4c demonstrates reduced displacement between point clouds using the Spherical Linear Interpolation (SLERP) model, though persistent horizontal drift remained apparent in the window frame.

**Figure 5:**
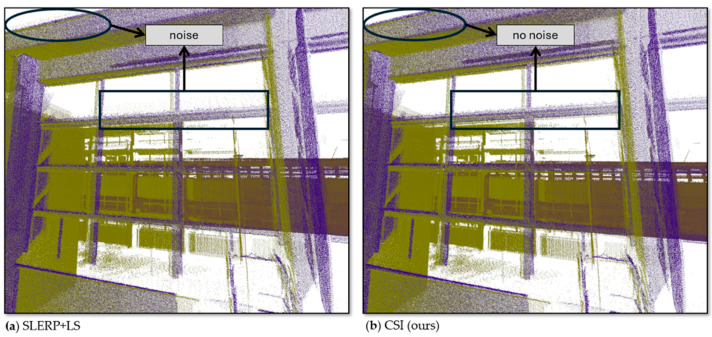
Magnified comparison of residual artifacts in the UFPR window frame. The blue cloud is from the beginning of the circuit, the green cloud is from the end of the circuit. (**a**) Misalignment artifacts remaining after SLERP + LS correction. (**b**) The cleaner, more accurate alignment provided by the proposed CSI model.

The SLERP + LS model in Figure 4d attenuated both horizontal and vertical drift, as evidenced by the sharper outlines of the windows and the ceiling line. However, it failed to correct one final cloud, which remained shifted upward and forward. This residual misalignment created subtle artifacts around the window arms. To provide a clearer view of this specific effect, Figure 5 presents a side-by-side, magnified comparison of this region from Figure 4d (SLERP + LS) and Figure 4e (CSI). We have included a video in the Supplementary Materials, where we highlight these changes in more detail.

**Figure 6:**
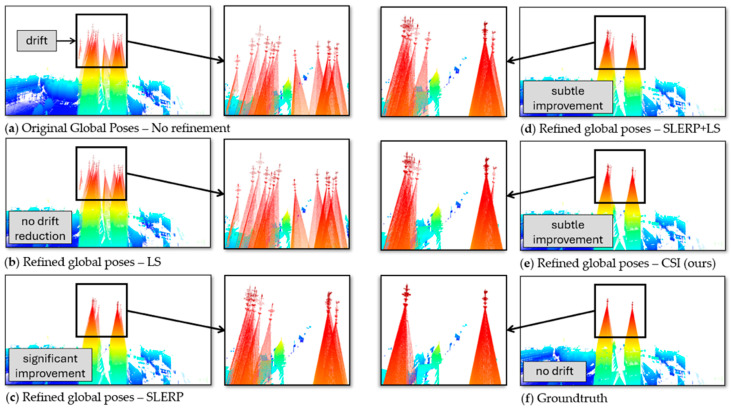
Effect of each GRM on drift correction in the Bremen dataset, with all scenes from the same viewpoint. The clouds have a gradient from blue to red in the positive direction of the Z-axis. The images show the accumulation of error on the two church tower spires. (**a**) **Original Poses (No Refinement)**: The accumulation of errors dispersed the tips of the two towers, making all 13 point clouds visible. (**b**) **LS**: Results in imperceptible changes, with all 13 clouds remaining dispersed. (**c**) **SLERP**: The rotation correction merged the clouds on the right tower into three, while the seven on the left became less dispersed. (**d**) **SLERP + LS**: A subtle, almost imperceptible change where one cloud on the right tower moved slightly closer to the others. (**e**) **CSI** (Ours): Provided a significant drift reduction, with only four crosses visible on the left and two on the right. (**f**) **Groundtruth**: The groundtruth poses showed no drift, with the towers appearing as a single, perfectly aligned cross.

In Figure 6, we performed the same analysis as with the Bremen dataset, but focused only on the top of the church towers, which, being far from the TLS positions, clearly demonstrated the effects of drift on the reconstruction of the building.

Figure 6a depicts the Bremen dataset reconstruction, with emphasis on the two church tower spires. Without global refinement in Figure 6a, all 13 dispersed point clouds were visible, evidenced by the 13 distinct crosses atop the towers. The application of the LS model in Figure 6b yielded no perceptible alignment improvements. Figure 6c demonstrates the effect of rotational correction via the SLERP model, which narrowed the spatial separation between towers.

In Figure 6d, the combined SLERP + LS model brought the clouds closer together. This change was subtle, but was noticeable when comparing the circles on the right side of Figure 6c,d. In Figure 6e, the CSI model reduced the drift a little more, although four scattered clouds were still noticeable in the left circle and two in the right circle. Finally, in Figure 6f, with the groundtruth poses, the towers came together precisely without drift.

These results, along with Table 2 and Table 3, suggest that circuits with greater drift errors are more likely to benefit from our CSI model. Our model not only reduces the magnitude of the error but also its variability, as indicated by the black lines on the error bars. This means that the CSI enhanced both the accuracy and stability of the global pose estimations more effectively than the other models. We also point out that in none of the five datasets did the CSI model deliver worse results than the original poses.

Although CSI performed worse than SLERP + LS in two datasets, it provides theoretical advantages through its dual quaternion formulation. These theoretical advantages translate into a more efficient implementation of the model, which allows for less execution time. Figure 6 compares the median execution times of the GRMs.

**Figure 7:**
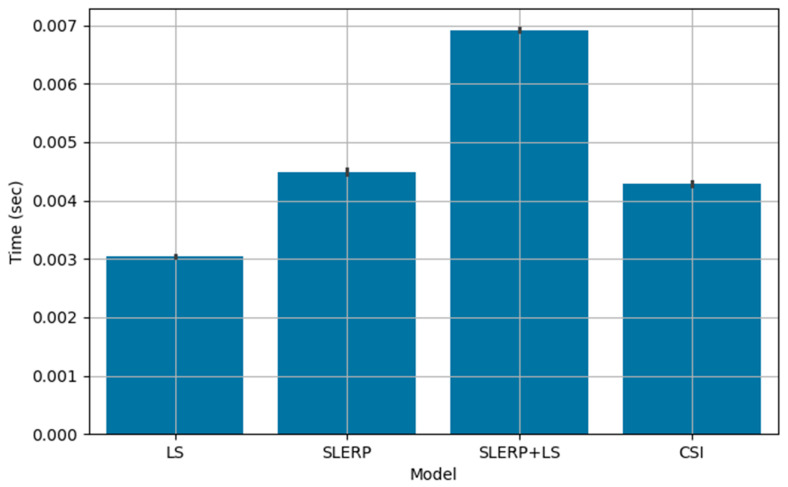
Execution time of each GRM. Median of one hundred tests across all datasets. Error bars use the Median Absolute Deviation (MAD).

Figure 7 demonstrates that CSI executed faster than both the SLERP and SLERP + LS models. All tests were implemented in Python 3.11 and run on our Intel i3 9350 KF CPU. This advantage stems from its reliance on the direct linear interpolation of dual quaternions. As detailed in [3], linear interpolation, followed by normalization, preserves the shortest path in dual quaternion space, the essential property for our application. By sacrificing constant rotational speed, CSI achieves increased computational efficiency, as it performs only one interpolation per pair of dual quaternions instead of the multiple interpolations typically utilized in animation.

CSI is based on direct linear interpolation (followed by normalization), which requires only basic arithmetic operations. This avoids the computationally more expensive trigonometric functions required by SLERP. Although SLERP only interpolates between quaternions and can benefit from similar optimization, its spherical interpolation process results in slower execution, as we use its default implementation.

## 4. Conclusions

This research introduced a linear Global Refinement Method (GRM) to correct drift errors in closed-loop trajectories from Terrestrial Laser Scanner (TLS) data. We derived a closed-form solution based on dual quaternion interpolation that simultaneously refines the translation and rotation of global poses. The formulation is non-iterative and does not require matrix decompositions.

Experimental evaluation on eight datasets demonstrated that the proposed method consistently improves the accuracy and stability of the global poses. The improvements were most significant in datasets with extensive trajectories. Furthermore, the GRM was computationally more efficient than other linear and decoupled approaches.

In summary, this work contributes to a unified model that reduces drift and enhances 3D reconstructions from 3D pose graphs. Future research will focus on extending the formulation to graphs containing multiple overlaps and generalizing the method to accommodate multiple cycles.

## Figures and Tables

**Table 1:** Datasets to compare the GRMs in drift correction.

Environment	Dataset	No. of Scans	Pts./Scan	Application	Mean Overlap	Mean Station Distance	TLS
Indoor	Theater	15	10 M	BIM	81%	7 m	Leica BLK 360
Outdoor	Crater	9	9 M	Environmental Surveillance	63%	6 m	Leica BLK 360
	Wood	11	15 M	Forestry	68%	8 m	Leica BLK 360
	UFPR	51	12 M	Urban Surveying	75%	6 m	Leica BLK 360
	Bremen	13	20 M	Urban Surveying	69%	39 m	Riegl VZ400
	Arch	5	26 M	Heritage Documentation	52%	17 m	Faro Focus 3D
	Facade	7	25 M	Urban Surveying	74%	4 m	Faro Focus 3D
	Courtyard	8	13 M	Archeology	79%	11 m	Faro Focus 3D

**Table 2:** Absolute error in meters of all models in all datasets. Total Error, MAE, and RMSE of the difference between groundtruth poses and estimated poses.

Dataset	Model	Total Error (m)	MAE (m)	RMSE (m)
Crater (n = 9 poses)	Original	0.035	0.004	0.005
	LS	0.162	0.018	0.022
	SLERP	0.072	0.008	0.011
	SLERP + LS	**0.018**	**0.002**	**0.002**
	CSI	0.033	0.004	0.005
Wood (n = 11 poses)	Original	0.256	0.023	0.029
	LS	0.282	0.026	0.032
	SLERP	0.241	0.022	0.029
	SLERP + LS	**0.112**	**0.010**	**0.012**
	CSI	0.189	0.017	0.024
Theater (n = 16 poses)	Original	0.236	0.015	0.018
	LS	0.308	0.019	0.023
	SLERP	0.329	0.021	0.024
	SLERP + LS	0.190	0.012	0.015
	CSI	**0.190**	**0.012**	**0.014**
Bremen (n = 13 poses)	Original	9.411	0.724	0.984
	LS	13.460	1.035	1.347
	SLERP	8.492	0.653	0.868
	SLERP + LS	8.744	0.673	0.928
	CSI	**5.563**	**0.428**	**0.591**
UFPR (n = 51 poses)	Original	24.685	0.484	0.605
	LS	34.890	0.684	0.807
	SLERP	31.795	0.623	0.745
	SLERP + LS	29.331	0.575	0.703
	CSI	**18.096**	**0.355**	**0.465**
Arch (n = 5 poses)	Original	0.216	0.043	0.062
	LS	0.212	0.043	0.071
	SLERP	0.155	0.031	0.045
	SLERP + LS	0.131	**0.026**	0.040
	CSI	**0.129**	**0.026**	**0.038**
Façade (n = 7 poses)	Original	0.078	0.011	0.014
	LS	0.077	0.011	0.013
	SLERP	0.077	0.011	0.014
	SLERP + LS	**0.076**	**0.011**	**0.013**
	CSI	**0.076**	**0.011**	**0.013**
Courtyard (n = 8 poses)	Original	1.063	0.133	0.172
	LS	0.636	0.079	0.094
	SLERP	1.021	0.128	0.165
	SLERP + LS	0.581	0.073	0.088
	CSI	**0.566**	**0.071**	**0.086**

**Table 3:** Relative error of each GRM about the original poses. Red means an error increase; blue means an error reduction.

Circuit/GRM	LS	SLERP	SLERP + LS	CSI
Crater	+367%	+108%	−49%	−5%
Wood	+10%	−6%	−56%	−26%
Theater	+31%	+40%	−19%	−20%
Bremen	+43%	−10%	−7%	−41%
UFPR	+41%	+29%	+19%	−27%
Arch	−1%	−28%	−39%	−40%
Façade	−1%	−1%	−2%	−2%
Courtyard	−40%	−4%	−45%	−47%
Average	+56%	+16%	−25%	−26%

## Data Availability

All of the code to reproduce results can be downloaded from: https://github.com/RubensBenevides/Closed-Form-Dual-Quaternion-Model-for-Drift-Correction-in-TLS-Pose-Circuits (accessed on 19 November 2025).

## Supplementary Materials

We provide a video containing a comparison of our model against the other three linear models discussed in the article. Video S1: “https://youtu.be/64euDcRq9VU (accessed on 19 November 2025), Drif correction: Comparing 4 linear models”.

## Abbreviations

The following abbreviations are used in this manuscript:

CSI	Constant Smooth Interpolation
GRM	Global Refinement Model
ICP	Iterative Closest Point
LS	Least Squares
SLERP	Spherical Linear Interpolation
TLS	Terrestrial Laser Scanner
UFPR	Universidade Federal do Paraná
